# Anti-diabetic effect of loganin by inhibiting FOXO1 nuclear translocation via PI3K/Akt signaling pathway in INS-1 cell

**DOI:** 10.22038/ijbms.2019.30246.7294

**Published:** 2019-03

**Authors:** Fang-Fang Mo, Hai-Xia Liu, Yi Zhang, Jing Hua, Dan-Dan Zhao, Tian An, Dong-Wei Zhang, Tian Tian, Si-Hua Gao

**Affiliations:** 1 Beijing University of Chinese Medicine, Beijing, 100029, China; 2 Beijing Open University, Beijing, 100081, China; 3 Beijing University of Chinese Medicine, Third Affiliated Hospital, Beijing, 100029, China

**Keywords:** Akt pathway, Factor forkhead box O1-(FOXO1), INS-1 cells, Loganin, Pancreatic β-cell

## Abstract

**Objective(s):**

JiangTangXiaoKe (JTXK) granule, a Chinese traditional herbal formula, has been clinically used and demonstrated to be beneficial in controlling high glucose and to relieve the symptoms of Type 2 diabetes mellitus patients for decades. In this study, we explored how loganin, one of the components in JTXK granule, mediated the anti-diabetic effect.

**Materials and Methods:**

We generate a cell model with the dysfunction of insulin secretion by over-expression FOXO1 in INS-1 cells. MTT method was used to detect cytotoxicity after treated with Loganin. ELISA analysis was used to examine insulin secretion. The expression levels of FOXO1 and Akt were evaluated by Western blot.

**Results:**

Treatment with Loganin did not change the expression level of FOXO1 in INS-1 cells, but increased phosphorylation of FOXO1 and inhibited the nuclear translocation and accumulation of FOXO1, which improved the insulin secretion of the cells. Mechanistically, we found PI3K/Akt signaling pathway involved in these effects, which were blocked by an Akt inhibitor, LY294002.

**Conclusion:**

Loganin mediated the subcellular distribution of FOXO1 via PI3K/Akt signaling pathway, which protected the function of insulin secretion in islet INS-1 cells.

## Introduction

Type 2 diabetes mellitus (T2DM) is a chronic metabolic disorder, mainly characterized by hyperglycemia and pancreatic β-cell dysfunction, leading to insufficient insulin secretion and insulin resistance (1, 2). Although it has long been accepted that insulin resistance is the leading factor in the pathogenesis of type 2 diabetes (3, 4), accumulating evidences over the past decades have showed that defects in pancreatic β-cell function is also one of major pathophysiologic abnormalities underlie most cases of T2DM (5-7). Pancreatic β-cell injury led to insufficient insulin supply that decreased sensitivity of the body tissues to respond to insulin (5-7). Therefore, rescuing the function of pancreatic β-cell in T2DM is crucial for the treatment of diabetes.

Forkhead box-containing protein o1 (FOXO1) is a key transcription factor in insulin signaling and metabolic homeostasis in response to oxidative stress (8). In pancreatic tissue, FOXO1 was specifically expressed in β-cell and was related to cell proliferation, differentiation, oxidative stress and apoptosis (9-12). In early phases of diabetes, FOXO1 is dephosphorylated and translocates into the nucleus. In late phases, FOXO1 disappears from β-cell as the insulin secretion decreases. This demonstrated FOXO1 nuclear translocation is an early sign of β-cell stress, and the distribution of FOXO1 may contribute to β-cell dedifferentiation and functional impairment (13, 14). In INS-1 cells, cytoplasmic phospho-FOXO1 was decreased and nuclear localization of FOXO1 was increased under proapoptotic and glucolipotoxic conditions (15), which inhibited cell proliferation and promoted cell apoptosis (10, 16). Therefore, inhibiting FOXO1 translocation into nucleus by increasing cytoplasmic FOXO1 phosphorylation is benefit to the function of INS-1 cells.

JiangtangXiaoke (JTXK) granule, a Chinese traditional herbal formula invented by ourselves previously, has been clinically use and demonstrated to be beneficial in controlling high glucose and to relieve the symptoms of T2DM patients for decades (17). Our previous studies have found that JTXK granule can improve glucose and lipid metabolism in clinical and animal models of diabetes mellitus (18, 19). However, the effective components of JTXK granule and the molecular mechanisms underlying anti-diabetic of JTXK granule remains unknown. The aim of the present study was to determine the effective components of JTXK granule and how they could regulate FOXO1 translocation to inhibit its transcriptional activity in INS-1 cell, and protect insulin secretion function. 

## Material and Methods

***Establishment of INS-1 cell line with high expression of FOXO1***

INS-1 cells were cultured in RPMI-1640 medium with 11.1 mM D-glucose supplemented with 10% FBS, 100 U/ml penicillin, 100 mg/ml streptomycin, 10 mM HEPES, 2 mM L-glutamine, 1 mM sodium pyruvate, and 50 mM β-mercaptoethanol. The culture medium was changed every 2 days. Cells were incubated at 37 ˚C in an atmosphere that supplemented with 5% CO_2_ and 95% air. 

The FOXO1 gene (NM_001191846) was PCR amplified and subcloned into GV308 expression vector using its AgeI/EcoRI restriction sites. Two synthetic oligonucleotide primers were designed to amplify the FOXO1 gene. The sequences for the primers were 

AACCGTCAGATCGCACCGGCGCCACCATGGCCGAAGCGCTC and TCCTTGTAGTCCATGAATTCGCCTGACACCCAGCTGTG

TGTTG. Lentivirus packaging and quality testing were performed in 293T cells. INS-1 cells were infected by Lentivirus containing FOXO1 gene, then following by puromycin creening. INS-1 cells with high expression a constitutively active form of FOXO1 were induced by 2.5 μg/ml doxycycline.

***Drug treatment***

INS-1 cells were seeded in 24-well plates at a density of 10000 cells per well. After 12 hr, 2.5 μg/ml doxycycline was added, and the cells were continuously cultured for 12 hr. Cells were cultured with or without loganin (Sigma), and with or without PI3K inhibitor for 24 hr. The PI3K inhibitor, LY294002, was dissolved in dimethyl sulfoxide as a 50 mM stock solution; the final concentration of LY294002 was 25 µM.

***Cell cytotoxicity analysis***

The viability of the cells was assessed by MTT assay, which is based on the reduction of MTT by the mitochondrial dehydrogenase of intact cells to a purple formazan product. Cells were seeded in 96-well plates at a density of 4000 cells per well. After 12 hrs, the cells were treated with different concentrations of Loganin for 24 hrs. After treatment, Loganin were carefully removed by aspiration. Hundered μl of 0.5 mg/ml MTT in cell culture medium was added to each well and incubated for 4 hrs. Hundered μl of DMSO was added to each well after 4 hrs. The absorbance was measured at 570 nm using a microplate reader (FLUOstar Omega, BMG LABIECH GmbH, GERMANY).

***ELISA Assay***

Insulin secretion was evaluated using ELISA assay, which was performed by ELISA kit (CUSABIO BIOTECH CO, LTD.). Briefly, 100 µl of the standards or samples of conditioned medium was plated into wells coated with antibody specific for rat insulin at 37 ˚C for 2 hrs, removed the liquid of each well and then incubated with 100 µl of a biotin-conjugated antibody reagent, at 37 ˚C for 1 hr. After incubation, the plates were washed three times and 100 µl of avidin conjugated Horseradish peroxidase (HRP) solution was added to each well and incubated for 1 hr at 37 ˚C. Following five further washes, TMB substrate solution (90 µl) was added to each well and the plate was allowed to develop at 37 ˚C in the dark. After 30 mins, 50 µl of stop solution was added and the absorbance of the samples was measured at 450 nm.

***Western blot analysis***

Total proteins were obtained from samples by cell lysis buffer for Western (Beyotime, Shanghai, China). Nuclear and cytoplasmic proteins were separated using Nuclear and Cytoplasmic Protein Extraction Kit (Beyotime) according to the manufacturer’s instructions. A total of 30 µg of protein samples was separated on a 10% SDS-polyacrylamide gel electrophoresis (PAGE) medium and transferred to polyvinylidene difluoride (PVDF) membranes (Millipore, Bedford, MA, USA). The membranes were blocked with 5% non-fat milk in Tris-buffered saline Tween-20 (TBST) for 1 hr at room temperature and incubated at 4 ˚C overnight with primary antibody. The Akt (Cell Signaling Technology), phospho-Akt (Thr308) (Cell Signaling Technology), FOXO1 (Cell Signaling Technology), phospho-FOXO1 (Ser256) (Abcam) and β-actin primary antibodies were at dilutions of 1:1000. After washing, the membranes were further incubated for 1 hr at room temperature with horseradish peroxidase conjugated secondary antibody. Then, the signal was visualized with high-sensitivity ECL luminous liquid and the images were captured using the Azure Bioimaging system (Azure C500; California, USA). The gray values of the blots were quantified using the Image Pro Plus 6.0 software, and normalized with the blots of corresponding β-actin as the internal control.

***Statistical analysis***

Data were expressed as mean±SD. Statistical analysis of data was performed by one-way ANOVA, followed by Student’s t-test. *P<0.05* was considered statistically significant.

## Results

***Dysfunction of insulin secretion after up-regulation of FOXO1 in INS-1 cells***

**Figure 1:**
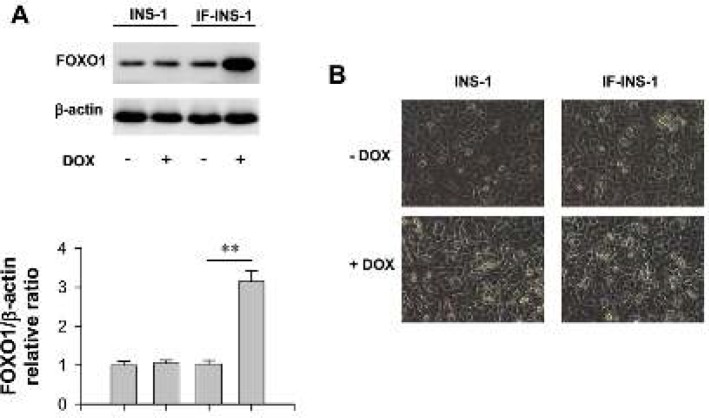
Up-regulation of FOXO1 in IF-INS-1 cells impaired insulin secretion function. (A) Western blot images for FOXO1 and β-actin, showing doxycycline (DOX) induced high expression of FOXO1 in IF-INS-1 cells, but not in INS-1 cells. (B) The morphological changes of cells were observed under high power microscope, and the cells do not vary greatly in size and shape among groups

We established an INS-1 cell line that can be induced to express a constitutively active form of FOXO1 by doxycycline (See methods). Here, this cell line was named as IF-INS-1. Western blot results showed that the expression level of FOXO1 was increased by about 1.5 fold after treatment the IF-INS-1 cells with 2.5 μg/ml doxycycline for 12 hrs (Figure 1A). In contrast, doxycycline has no effect on the expression level of FOXO1 in INS-1 cells (Figure 1A). Notably, doxycycline treatment did not change the morphology of cells (Figure 1B), indicating that doxycycline itself and high expression of FOXO1 has no effect on the cell size and shape. 

**Figure 2:**
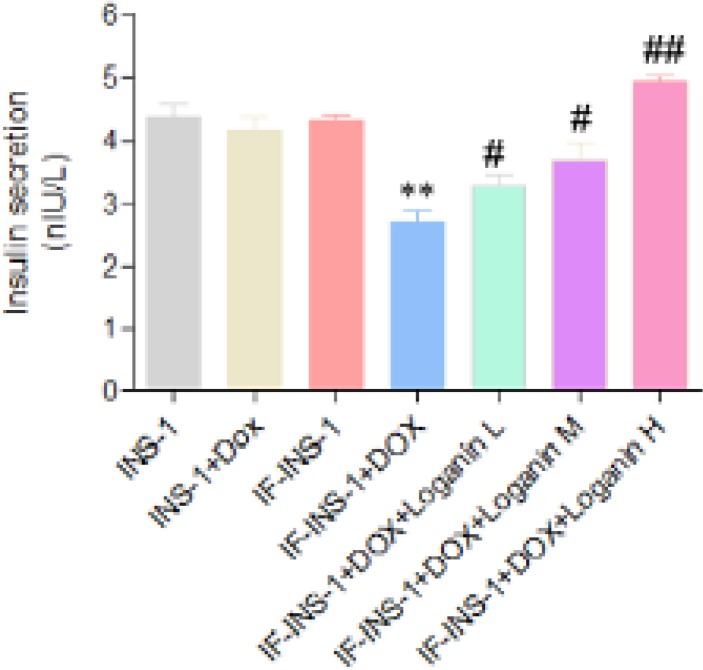
Loganin rescued the dysfunction of insulin secretion in IF-INS-1 cells induced by high expression of FOXO1. **P<0.01 vs IF-INS group. #*P<*0.05, ##*P<*0.01 vs IF-INS -DOX group

Next, we used ELISA assay to detect the insulin secretion function of the cells. As shown in Figure 2, the insulin content in the medium of INS-1 and IF-INS-1 cells before doxycycline treatment was comparable. After 2.5 μg/ml doxycycline treatment, the insulin secretion was dramatically decreased in IF-INS-1 cells (Figure 2), indicating that high expression of FOXO1 in IF-INS-1 cells impaired the insulin secretion function. 

***Loganin protected insulin secretion function by regulating FOXO1 translocation***

To investigate whether loganin, one of the components of JTXK granule, mediated the anti-diabetic effect of JTXK granule, we treated the IF-INS-1 cells with 2.5 μg/ml doxycycline for 12 hrs, following with low, medium and high concentrations (0.2, 0.6, and 2 µM) of loganin for 24 hrs. ELISA assay showed that the dysfunction of insulin secretion induced by high expression of FOXO1 was rescued by loganin treatment in a does-dependent manner (Figure 2). High concentration of loganin treatment totally rescued the dysfunction of insulin secretion in IF-INS-1 cells. 

**Figure 3:**
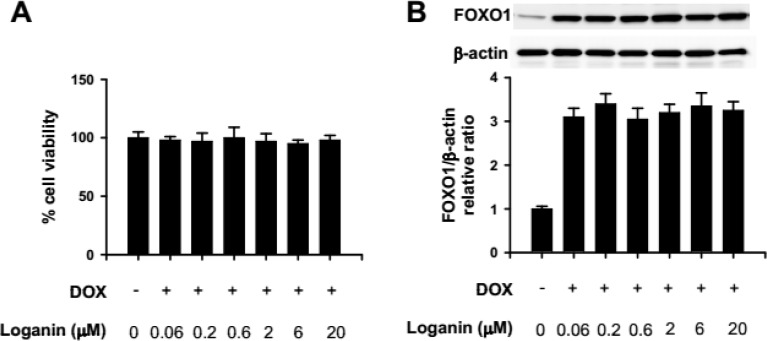
Loganin had no toxicity effect on IF-INS-1 cells and did not change the expression level of FOXO1. (A) Cell viability was measured by MTT assay after different concentrations of loganin treatment. (B) Western blot result showing Loganin treatment did not change the expression level of FOXO1

Next, we explored how loganin exerts the anti-diabetic effect in IF-INS-1 cells. The cells were treated as above, 2.5 μg/ml doxycycline for 12 hrs, following with different concentrations of loganin for 24 hrs. Firstly, to make sure loganin has no cytotoxicity to the IF-INS-1 cells, we used MTT assay to evaluate the viability of the cells, and found treatment with 0.06, 0.2, 0.6, 2, 6, 20 µM loganin has no effect on cell viability (Figure 3A). Western blot was used to check the expression level of FOXO1. We found on the change in FOXO1 expression after treatment with different concentrations of loganin (Figure 3B). In the following experiment, we chose 2 µM loganin to treat the cells.

**Figure 4:**
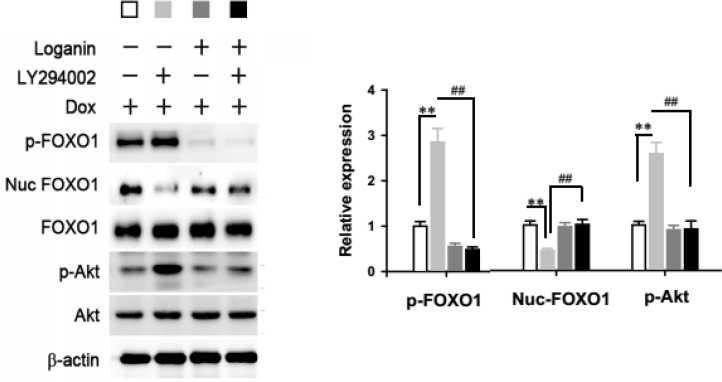
Loganin mediated the nuclear exclusion of FOXO1 by phosphorylation via PI3K/Akt signaling pathway. Western blot detecting the levels of phosphorylated FOXO1 (p-FOXO1), FOXO1 in the nuclear (nuc-FOXO1), total FOXO1, phosphorylated Akt (p-Akt) and total Akt

The transcriptional activity of FOXO1 relies on the nuclear translocation of dephosphorylated form of FOXO1 from cytoplasm. Next, we detected the levels of phosphorylated form of FOXO1 after loganin treatment. Interestingly, although 2 µM loganin treatment did not change the total protein level of FOXO1, it significantly increased the phosphorylation of FOXO1 (Figure 4), which would promote the translocation of FOXO1 from cytoplasm to nuclear. This observation was consistent with the results that the protein level of FOXO1 in the nuclear was markedly decreased after 2 µM loganin treatment (Figure 4). 

***Anti-diabetic effect of loganin was mediated by PI3K/Akt signaling pathway***

Phosphorylation of FOXO1 is mediation by PI3K/Akt signaling pathway. Namely, the activation of PI3K phosphorylates Akt. Akt then phosphorylates FOXO1, causing nuclear exclusion. As shown in Figure 4, loganin treatment also elevated the phosphorylated Akt, but not changed the level of total protein, indicating that PI3K/Akt signaling pathway may involve in the effect of loganin.

**Figure 5:**
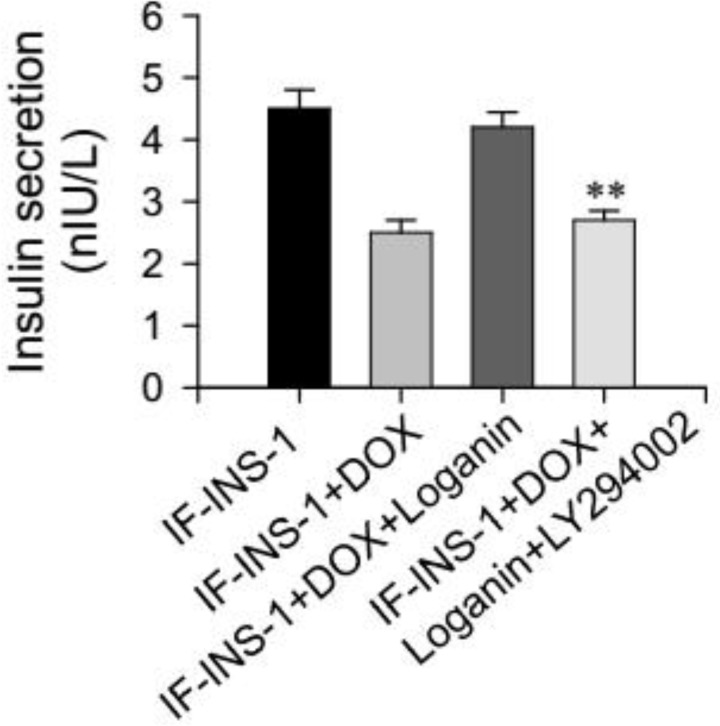
Anti-diabetic effect of loganin was blockedd by Akt inhibitor, LY294002. **P<0.01 vs IF-INS+DOX+Loganin group

To test this, here we used LY294002, an Akt inhibitor, to block the Akt signaling during the treatment of loganin. We found that in IF-INS-1 cells, the increased phosphorylatd FOXO1 level and nuclear accumulation of FOXO1 induced by loganin were fully blocked by LY294002 treatment (Figure 4). As expected, the function of insulin secretion can not be rescued by loganin treatment in the presence of LY294002 (Figure 5). These results suggest that PI3K/Akt/FOXO1 signaling pathway mediated the effect of loganin on FOXO1 phosphorylation and nuclear exclusion.

## Discussion

In the present study, using a high expression of FOXO1 cell line we demonstrated that loganin mediated the subcellular distribution of FOXO1 by increasing FOXO1 phosphrylation, then regulating the insulin secretion function. This process was medicated by PI3K/Akt/FOXO1 signaling pathway, which may provide a potential strategy to rescue the function of pancreatic β-cell in T2DM.

FOXO1 is a double-edged sword that can inhibit β-cell proliferation by inhibiting expression of pancreatic and duodenum homeobox 1 and protect against β-cell failure induced by oxidative stress through neurogenic differentiation 1 and muscyloaponeurotic fibrosarcoma oncogene homolog A induction (11). Previous reports have shown that the expression of FOXO1 mRNA in pancreatic islet of T2DM patients was significantly higher than that of non-diabetic control group (20). In animal studies, overexpression of FOXO1 in both the hypothalamus and pancreas causes obesity, glucose intolerance and decreased insulin sensitivity (21). In contrast, low expression of FOXO1 increased insulin sensitivity *in vivo* and* in vitro *(22). Therefore, down-regulation of FOXO1 in T2DM may be a new strategy for the treatment of diabetes mellitus. 

It has been shown that the JTXK granule has a protective effect on islets. Previous studies have shown that JTXKS can inhibit apoptosis of INS-1 cells and protect islet cell function (23). However, the effective components and the underlying mechanism is still unclear. After treating the INS-1 cells and isolated rat islets with glucotoxic conditions, phosphorylation of FOXO1 at Ser-256 was significantly decreased, which led to the nuclear accumulation of FOXO1 (24, 25). Similarly, under the condition of lipotoxicity and overnutrition, the transcriptional activity of FOXO1 was increased (26). In INS-1 cells with high expression of FOXO1, we found the level of phosphorylated FOXO1 was low and the FOXO1 mainly located in the nuclear, which increased the transcriptional activity of FOXO1 and impaired the insulin secretion function. Interestingly, Loganin treatment increased phosphorylation of FOXO1 and inhibited the nuclear accumulation of FOXO1, which can be blocked by LY294002. 

## Conclusion

We demonstrated that loganin mediated the nuclear exclusion of FOXO1 by phosphorylation via PI3K/Akt signaling pathway, which protected the function of insulin secretion in islet INS-1 cells.
